# Supplementary material for: Visible light reduces C. elegans longevity
Source: Nat Commun. 2018 Mar 2;9:927. doi: 10.1038/s41467-018-02934-5 (PMC5834526; doi:10.1038/s41467-018-02934-5)
Supplement: Supplementary file 1 — Supplementary Information [file 41467_2018_2934_MOESM1_ESM.pdf]

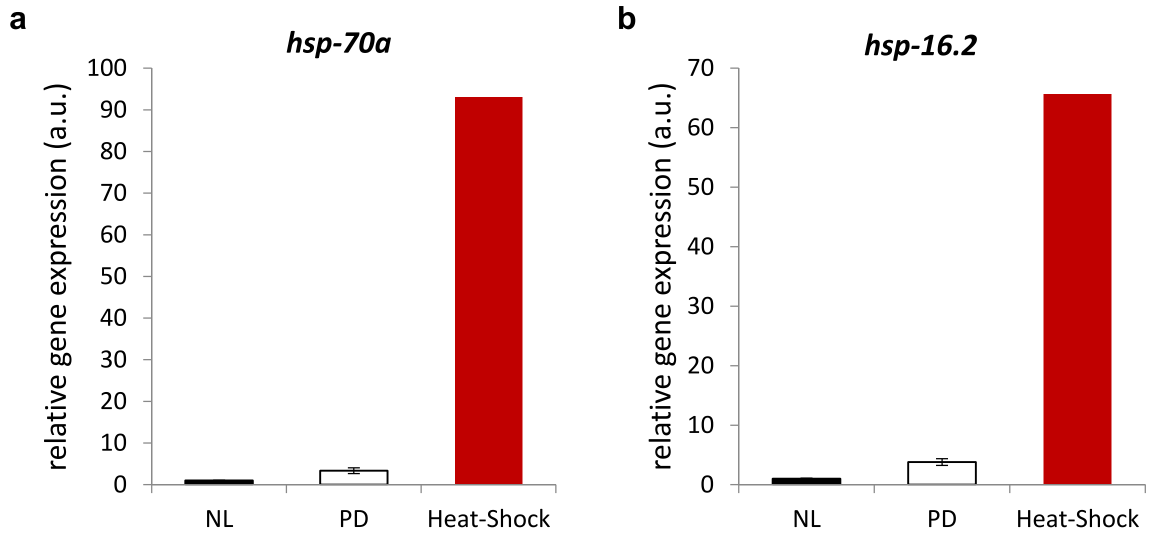

Supplementary Figure 1: Light exposure is not creating a heat stress to worms. Relative gene expression levels for hsp-70a and hsp-16.2 in worms exposed to permanent day (PD) or no light (NL) condition from hatch and harvested at D1 adult, versus D1 adult worms raised under NL condition and exposed to 37°C for 1 hour at D1 adult, and harvested 8 hours after the heat stress.

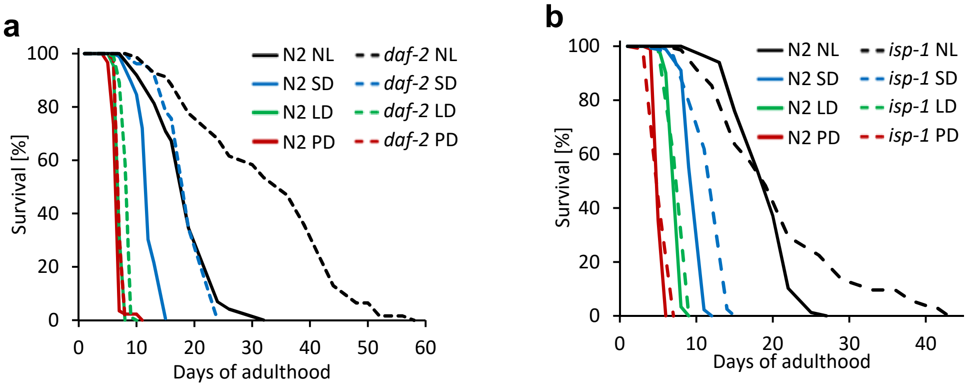

<u>Supplementary Figure 2:</u> Repeats of the lifespans shown in Figure 6a and b showing that increasing defense mechanisms protects *C. elegans* from light toxicity. **a**, lifespan of *daf-2*(e1370) mutants versus N2 worms under PD, LD, SD, and NL photoperiods. **b**, lifespan of *isp-1*(qm150) mutants versus N2 worms under PD, LD, SD, and NL photoperiods.

Supplementary Table 1: Statistics for lifespan experiments performed in this study

Strain-Condition	Mean lifespan ± SEM (days)	Median (days)	Total #animals died/total	*P* - Value							
**Figure 1**											
**panel b**											
				N2 - NL	N2 - SD	N2 - LD	N2 - PD				
N2 - NL	14.7±0.5	16	61/92	-	< 0.0001	< 0.0001	< 0.0001				
N2 - SD	7.9±0.1	7	89/100	< 0.0001	-	< 0.0001	< 0.0001				
N2 - LD	6.3±0.1	7	94/100	< 0.0001	< 0.0001	-	< 0.0001				
N2 - PD	4.8±0.1	5	87/100	< 0.0001	< 0.0001	< 0.0001	-				
**panel c**											
				JU361 - NL	JU361 - SD	JU361 - LD	JU361 - PD				
JU361 - NL	21.5±0.3	21	63/100	-	< 0.0001	< 0.0001	< 0.0001				
JU361 - SD	15.2±0.2	16	61/100	< 0.0001	-	< 0.0001	< 0.0001				
JU361 - LD	9.7±0.1	9	83/100	< 0.0001	< 0.0001	-	< 0.0001				
JU361 - PD	6.5±0.1	6	70/100	< 0.0001	< 0.0001	< 0.0001	-				
**panel d**											
				NL heat							
NL heat	28.2±0.4	29	26/100	-							
PD heat	5.7±0.1	6	36/100	< 0.0001							
**panel e**											
				NL kanamycin							
NL kanamycin	19.3±0.7	24	47/100	-							
PD kanamycin	5.7±0.1	6	86/100	< 0.0001							
**panel f**											
				NL plates							
NL plates	9.5±0.3	10	78/100	-							
PD plates	9.3±0.2	10	81/100	0.419							
**Figure 2**											
**panel d**											
				N2 - NL	N2 - SD	N2 - LD	N2 - PD				
N2 - NL	18.2±0.4	19	80/100	-	< 0.0001	< 0.0001	< 0.0001				
N2 - SD	9.0±0.1	10	89/100	< 0.0001	-	< 0.0001	< 0.0001				
N2 - LD	6.8±0.1	7	90/100	< 0.0001	< 0.0001	-	< 0.0001				
N2 - PD	4.3±0.1	4	96/100	< 0.0001	< 0.0001	< 0.0001	-				
**Figure 3**											
**panel a**											
				N2 - NL	*lite-1(ok530)* - NL	N2 - PD	*lite-1(ok530)* - PD				
N2 - NL	17.0±0.4	19	66/100	-	0.778	< 0.0001	< 0.0001				
lite-1(ok530) - NL	17.2±0.4	19	68/100	0.778	-	< 0.0001	< 0.0001				
N2 - PD	5.7±0.1	5	82/100	< 0.0001	< 0.0001	-	< 0.0001				
lite-1(ok530) - PD	5.1±0.1	5	90/100	< 0.0001	< 0.0001	< 0.0001	-				
**panel b**											
				N2 - NL	*lite-1(xu7)* - NL	*lite-1(ce314)* - NL	N2 - PD	*lite-1(xu7)* - PD	*lite-1(ce314)* - PD		
N2 - NL	16.1±0.5	18	99/100	-	< 0.05	0.290	< 0.0001	< 0.0001	< 0.0001		
lite-1(xu7) - NL	15.7±0.4	18	99/100	< 0.05	-	0.056	< 0.0001	< 0.0001	< 0.0001		
lite-1(ce314) - NL	16.4±0.4	18	97/100	0.290	0.056	-	< 0.0001	< 0.0001	< 0.0001		
N2 - PD	4.0±0.0	4	99/100	< 0.0001	< 0.0001	< 0.0001	-	0.314	1.000		
lite-1(xu7) - PD	4.0±0.0	4	100/100	< 0.0001	< 0.0001	< 0.0001	0.314	-	0.314		
lite-1(ce314) - PD	4.0±0.0	4	100/100	< 0.0001	< 0.0001	< 0.0001	1.000	0.314	-		
**panel c**											
				N2 - NL	*tax-2(p671)* - NL	*tax-2(p691)* - NL	*tax-2(p694)* - NL	N2 - PD	*tax-2 (p671)* - PD	*tax-2 (p691)* - PD	*tax-2 (p694)* - PD
N2 - NL	17.0±0.4	19	66/100	-	0.237	< 0.001	< 0.05	< 0.0001	< 0.0001	< 0.0001	< 0.0001
tax-2(p671) - NL	18.9±0.6	19	63/102	0.237	-	< 0.0001	0.281	< 0.0001	< 0.0001	< 0.0001	< 0.0001
tax-2(p691) - NL	15.0±0.5	18	57/100	< 0.001	< 0.0001	-	< 0.0001	< 0.0001	< 0.0001	< 0.0001	< 0.0001
tax-2(p694) - NL	18.1±0.5	19	69/90	< 0.05	0.281	< 0.0001	-	< 0.0001	< 0.0001	< 0.0001	< 0.0001
N2 - PD	5.7±0.1	5	82/100	< 0.0001	< 0.0001	< 0.0001	< 0.0001	-	0.217	0.336	< 0.0001
tax-2 (p671) - PD	5.9±0.1	5	87/100	< 0.0001	< 0.0001	< 0.0001	< 0.0001	0.217	-	< 0.0001	< 0.0001
tax-2 (p691) - PD	5.5±0.1	6	76/94	< 0.0001	< 0.0001	< 0.0001	< 0.0001	0.336	< 0.0001	-	< 0.0001
tax-2 (p694) - PD	5.1±0.1	5	69/90	< 0.0001	< 0.0001	< 0.0001	< 0.0001	< 0.0001	< 0.0001	< 0.0001	-
**panel d**											
				N2 - NL	N2 - low	N2 - moderate	N2 - high				
N2 - NL	13.8±0.5	17	42/100	-	< 0.0001	< 0.0001	< 0.0001				
N2 - low	7.4±0.1	8	84/100	< 0.0001	-	< 0.0001	< 0.0001				
N2 - moderate	5.4±0.1	5	76/100	< 0.0001	< 0.0001	-	< 0.0001				
N2 - high	2.1±0.0	2	98/100	< 0.0001	< 0.0001	< 0.0001	-				
**panel f**											
				N2 - no light							
N2 - no light	14.6±0.5	15	91/100	-							
N2 - bench light	10.9±0.4	10	75/100	< 0.0001							

panel g

				N2 - dark room
N2 - dark room	12.5±0.4	11	81/100	-
N2 - bench	11.0±0.3	11	73/100	< 0.01

Figure 4

panel c

				N2 - NL	N2 - SD	N2 - LD	N2 - PD
N2 - NL	17.0±0.5	16	89/100	-	0.294	0.937	0.950
N2 - SD	16.4±0.5	16	78/100	0.294	-	0.211	0.227
N2 - LD	17.3±0.4	16	84/100	0.937	0.211	-	0.928
N2 - PD	17.3±0.4	16	70/89	0.950	0.227	0.928	-

panel d

				NL
NL	19.4±0.8	18	65/100	-
Blue light	6.9±0.2	7	53/100	< 0.0001

panel e

				NL
NL	13.4±0.5	15	53/96	-
Green light	8.7±0.3	8	63/100	< 0.0001

panel f

				NL
NL	14.0±0.3	13	59/91	-
Yellow light	11.3±0.4	11	75/99	< 0.0001

panel g

				NL
NL	15.6±0.4	16	76/100	-
Red light	13.2±0.4	12	76/100	< 0.001

Figure 6

panel a

				N2 - NL	N2 - SD	N2 - LD	N2 - PD	*daf-2(e1370)* - NL	*daf-2(e1370)* - SD	*daf-2(e1370)* - LD	*daf-2(e1370)* - PD
N2 - NL	18.6±0.4	19	98/100	-	< 0.0001	< 0.0001	< 0.0001	< 0.0001	< 0.0001	< 0.0001	< 0.0001
N2 - SD	10.2±0.2	10	93/100	< 0.0001	-	< 0.0001	< 0.0001	< 0.0001	< 0.0001	< 0.0001	< 0.0001
N2 - LD	6.0±0.1	6	98/100	< 0.0001	< 0.0001	-	< 0.0001	< 0.0001	< 0.0001	< 0.0001	< 0.0001
N2 - PD	4.4±0.1	4	99/100	< 0.0001	< 0.0001	< 0.0001	-	< 0.0001	< 0.0001	< 0.0001	< 0.0001
daf-2(e1370) - NL	41.5±1.1	44	96/100	< 0.0001	< 0.0001	< 0.0001	< 0.0001	-	-	< 0.0001	< 0.0001
daf-2(e1370) - SD	19.0±0.4	18	92/100	< 0.0001	< 0.0001	< 0.0001	< 0.0001	< 0.0001	-	< 0.0001	< 0.0001
daf-2(e1370) - LD	11.3±0.2	12	75/80	< 0.0001	< 0.0001	< 0.0001	< 0.0001	< 0.0001	< 0.0001	-	< 0.0001
daf-2(e1370) - PD	7.8±0.1	8	79/80	< 0.0001	< 0.0001	< 0.0001	< 0.0001	< 0.0001	< 0.0001	< 0.0001	-

panel b

				N2 - NL	N2 - SD	N2 - LD	N2 - PD	*isp-1(qm150)* - NL	*isp-1(qm150)* - SD	*isp-1(qm150)* - LD	*isp-1(qm150)* - PD
N2 - NL	17.3±0.6	17	95/100	-	< 0.0001	< 0.0001	< 0.0001	< 0.0001	< 0.0001	< 0.0001	< 0.0001
N2 - SD	13.3±0.2	15	96/100	< 0.0001	-	< 0.0001	< 0.0001	< 0.0001	< 0.0001	< 0.0001	< 0.0001
N2 - LD	5.6±0.1	6	100/100	< 0.0001	< 0.0001	-	< 0.0001	< 0.0001	< 0.0001	< 0.0001	< 0.0001
N2 - PD	4.8±0.0	5	99/100	< 0.0001	< 0.0001	< 0.0001	-	< 0.0001	< 0.0001	< 0.0001	< 0.0001
isp-1(qm150) - NL	25.0±0.5	25	88/100	< 0.0001	< 0.0001	< 0.0001	< 0.0001	-	< 0.0001	< 0.0001	< 0.0001
isp-1(qm150) - SD	15.2±0.1	15	96/100	< 0.0001	< 0.0001	< 0.0001	< 0.0001	< 0.0001	-	< 0.0001	< 0.0001
isp-1(qm150) - LD	7.0±0.1	7	92/100	< 0.0001	< 0.0001	< 0.0001	< 0.0001	< 0.0001	< 0.0001	-	< 0.0001
isp-1(qm150) - PD	6.1±0.1	6	99/100	< 0.0001	< 0.0001	< 0.0001	< 0.0001	< 0.0001	< 0.0001	< 0.0001	-

panel c

				SD+Vehicle	SD+NAC low	SD+NAC high	SD+VitC low	SD+VitC high
SD+Vehicle	11.1±0.2	11	97/100	-	< 0.0001	< 0.0001	< 0.0001	< 0.0001
SD+NAC low	15.6±0.2	15	92/98	< 0.0001	-	0.781	0.454	< 0.0001
SD+NAC high	15.7±0.1	15	93/99	< 0.0001	0.781	-	0.264	< 0.0001
SD+VitC low	15.9±0.2	16	74/99	< 0.0001	0.454	0.264	-	< 0.001
SD+VitC high	16.9±0.2	16	91/99	< 0.0001	< 0.0001	< 0.0001	< 0.001	-

panel d

				BL+Vehicle	BL+VitC low	BL+VitC high	BL+NAC low	BL+NAC high	NL+Vehicle
BL+Vehicle	9.9±0.1	9	97/100	-	< 0.0001	< 0.0001	< 0.0001	< 0.0001	< 0.0001
BL+VitC low	17.3±0.3	18	100/100	< 0.0001	-	< 0.0001	< 0.0001	< 0.0001	< 0.0001
BL+VitC high	15.0±0.5	16	97/100	< 0.0001	< 0.0001	-	< 0.0001	< 0.0001	< 0.01
BL+NAC low	12.9±0.2	12	96/100	< 0.0001	< 0.0001	< 0.0001	-	< 0.01	< 0.01
BL+NAC high	20.0±0.3	21	90/100	< 0.0001	< 0.0001	< 0.0001	< 0.01	-	< 0.0001
NL+Vehicle	17.9±0.5	21	99/100	< 0.0001	< 0.0001	< 0.01	< 0.01	< 0.0001	-

Replicates

Replicates for Figure 1

panel b

				N2 - NL	N2 - LD	N2 - PD
N2 - NL	15.7±0.4	15	71/100	-	< 0.0001	< 0.0001
N2 - LD	5.9±0.1	6	92/100	< 0.0001	-	< 0.0001
N2 - PD	4.2±0.1	4	79/100	< 0.0001	< 0.0001	-

panel f

				NL plates
NL plates	12.2±0.5	12	74/100	-
PD plates	12.0±0.5	12	62/100	0.944

Replicates for Figure 2

panel d

				N2 - NL	N2 - SD	N2 - LD	N2 - PD
N2 - NL	17.2±0.6	17	82/98	-	< 0.0001	< 0.0001	< 0.0001
N2 - SD	11.2±0.2	11	79/100	< 0.0001	-	< 0.0001	< 0.0001
N2 - LD	6.3±0.1	6	88/100	< 0.0001	< 0.0001	-	< 0.0001
N2 - PD	5.8±0.1	6	85/100	< 0.0001	< 0.0001	< 0.0001	-

				N2 - NL	N2 - SD	N2 - LD	N2 - PD
N2 - NL	13.7±0.4	12	72/100	-	0.412	< 0.0001	< 0.0001
N2 - SD	13.9±0.3	16	42/101	0.412	-	< 0.0001	< 0.0001
N2 - LD	7.8±0.1	8	96/101	< 0.0001	< 0.0001	-	< 0.0001
N2 - PD	5.4±0.1	5	89/100	< 0.0001	< 0.0001	< 0.0001	-

				N2 - NL	N2 - SD	N2 - LD	N2 - PD
N2 - NL	17.3±0.6	17	95/100	-	< 0.0001	< 0.0001	< 0.0001
N2 - SD	13.3±0.2	15	96/100	< 0.0001	-	< 0.0001	< 0.0001
N2 - LD	5.6±0.1	6	100/100	< 0.0001	< 0.0001	-	< 0.0001
N2 - PD	4.8±0.0	5	99/100	< 0.0001	< 0.0001	< 0.0001	-

Replicates for Figure 3

panel a

				N2 - NL	*lite-1(ok530)* - NL	N2 - PD	*lite-1(ok530)* - PD
N2 - NL	18.2±0.4	19	80/100	-	< 0.0001	< 0.0001	< 0.0001
lite-1(ok530) - NL	20.6±0.5	22	66/100	< 0.0001	-	< 0.0001	< 0.0001
N2 - PD	4.3±0.1	4	96/100	< 0.0001	< 0.0001	-	< 0.0001
lite-1(ok530) - PD	5.0±0.0	5	43/100	< 0.0001	< 0.0001	< 0.0001	-

panel b

				N2 - PD	*lite-1(xu7)* - PD	*lite-1(ce314)* - PD
N2 - PD	4.0±0.0	4	98/100	-	< 0.0001	0.052
lite-1(xu7) - PD	3.7±0.0	4	99/100	< 0.0001	-	< 0.05
lite-1(ce314) - PD	3.9±0.1	4	100/100	0.052	< 0.05	-

panel f

				N2 - no light
N2 - no light	13.6±0.5	15	52/100	-
N2 - bench light	11.3±0.4	11	47/97	< 0.0001

				N2 - no light
N2 - no light	13.4±0.6	14	65/100	-
N2 - bench light	11.1±0.4	11	75/100	< 0.001

panel g

				N2 - dark room
N2 - dark room	11.1±0.3	10	67/100	-
N2 - bench	10.1±0.3	8	80/100	< 0.05

Replicates for Figure 4

panel c

				NL
NL	19.2±0.6	20	77/100	-
Blue light	7.3±0.1	7	63/100	< 0.0001

panel d

				NL
NL	14.4±0.5	16	69/100	-
Green light	7.8±0.3	8	76/100	< 0.0001

panel f

				NL
NL	16.0±0.4	15	81/101	-
Red light	14.1±0.4	15	83/100	< 0.01

Replicates for Figure 6

panel a

				N2 - NL	N2 - SD	N2 - LD	N2 - PD	*daf-2(e1370)* - NL	*daf-2(e1370)* - SD	*daf-2(e1370)* - LD	*daf-2(e1370)* - PD
N2 - NL	17.2±0.6	17	82/98	-	< 0.0001	< 0.0001	< 0.0001	< 0.0001	< 0.01	< 0.0001	< 0.0001
N2 - SD	11.2±0.2	11	79/100	< 0.0001	-	< 0.0001	< 0.0001	< 0.0001	< 0.0001	< 0.0001	< 0.0001
N2 - LD	6.3±0.1	6	88/100	< 0.0001	< 0.0001	-	< 0.0001	< 0.0001	< 0.0001	< 0.0001	0.808
N2 - PD	5.8±0.1	6	85/100	< 0.0001	< 0.0001	< 0.0001	-	< 0.0001	< 0.0001	< 0.0001	< 0.0001
daf-2(e1370) - NL	32.5±1.5	35	63/100	< 0.0001	< 0.0001	< 0.0001	< 0.0001	-	< 0.0001	< 0.0001	< 0.0001
daf-2(e1370) - SD	18.5±0.5	18	73/101	< 0.01	< 0.0001	< 0.0001	< 0.0001	< 0.0001	-	< 0.0001	< 0.0001
daf-2(e1370) - LD	7.5±0.1	8	89/100	< 0.0001	< 0.0001	< 0.0001	< 0.0001	< 0.0001	< 0.0001	-	< 0.0001
daf-2(e1370) - PD	6.3±0.1	6	89/100	< 0.0001	< 0.0001	0.808	< 0.0001	< 0.0001	< 0.0001	< 0.0001	-

				N2 - SD	N2 - PD	*daf-2(e1370)* - SD	*daf-2(e1370)* - PD
N2 - SD	10.7±0.3	12	93/100	-	< 0.0001	< 0.0001	< 0.0001
N2 - PD	4.4±0.1	4	95/100	< 0.0001	-	< 0.0001	< 0.0001
daf-2(e1370) - SD	21.4±0.4	23	99/100	< 0.0001	< 0.0001	-	< 0.0001
daf-2(e1370) - PD	6.9±0.1	7	96/100	< 0.0001	< 0.0001	< 0.0001	-

**panel b**											
				N2 - SD	N2 - LD	N2 - PD	*isp-1(qm150)* - SD	*isp-1(qm150)* - LD	*isp-1(qm150)* - PD		
N2 - SD	10.2±0.2	10	93/100	-	< 0.0001	< 0.0001	< 0.0001	< 0.0001	< 0.0001		
N2 - LD	6.0±0.1	6	98/100	< 0.0001	-	< 0.0001	< 0.0001	< 0.01	< 0.0001		
N2 - PD	4.4±0.1	4	99/100	< 0.0001	< 0.0001	-	< 0.0001	< 0.0001	< 0.0001		
isp-1(qm150) - SD	11.6±0.2	12	89/90	< 0.0001	< 0.0001	< 0.0001	-	< 0.0001	< 0.0001		
isp-1(qm150) - LD	6.3±0.1	6	85/90	< 0.0001	< 0.01	< 0.0001	< 0.0001	-	< 0.0001		
isp-1(qm150) - PD	5.5±0.1	5	86/90	< 0.0001	< 0.0001	< 0.0001	< 0.0001	< 0.0001	-		
				N2 - NL	N2 - SD	N2 - LD	N2 - PD	*isp-1(qm150)* - NL	*isp-1(qm150)* - SD	*isp-1(qm150)* - LD	*isp-1(qm150)* - PD
N2 - NL	18.2±0.4	19	80/100	-	< 0.0001	< 0.0001	< 0.0001	0.370	< 0.0001	< 0.0001	< 0.0001
N2 - SD	9.0±0.1	10	89/100	< 0.0001	-	< 0.0001	< 0.0001	< 0.0001	< 0.0001	< 0.0001	< 0.0001
N2 - LD	6.8±0.1	7	90/100	< 0.0001	< 0.0001	-	< 0.0001	< 0.0001	< 0.0001	< 0.0001	< 0.0001
N2 - PD	4.3±0.1	4	96/100	< 0.0001	< 0.0001	< 0.0001	-	< 0.0001	< 0.0001	< 0.0001	< 0.0001
isp-1(qm150) - NL	18.2±0.8	18	54/91	0.370	< 0.0001	< 0.0001	< 0.0001	-	< 0.0001	< 0.0001	< 0.0001
isp-1(qm150) - SD	11.4±0.3	13	79/100	< 0.0001	< 0.0001	< 0.0001	< 0.0001	< 0.0001	-	< 0.0001	< 0.0001
isp-1(qm150) - LD	7.2±0.1	8	81/100	< 0.0001	< 0.0001	< 0.0001	< 0.0001	< 0.0001	< 0.0001	-	< 0.0001
isp-1(qm150) - PD	4.8±0.1	4	88/100	< 0.0001	< 0.0001	< 0.0001	< 0.0001	< 0.0001	< 0.0001	< 0.0001	-
				N2 - NL	N2 - SD	N2 - PD	*isp-1(qm150)* - NL	*isp-1(qm150)* - SD	*isp-1(qm150)* - PD		
N2 - NL	18.6±0.4	19	98/100	-	< 0.0001	< 0.0001	< 0.0001	< 0.0001	< 0.0001		
N2 - SD	10.7±0.3	12	93/100	< 0.0001	-	< 0.0001	< 0.0001	0.845	< 0.0001		
N2 - PD	4.4±0.1	4	95/100	< 0.0001	< 0.0001	-	< 0.0001	< 0.0001	0.058		
isp-1(qm150) - NL	23.8±0.6	23	82/90	< 0.0001	< 0.0001	< 0.0001	-	< 0.0001	< 0.0001		
isp-1(qm150) - SD	10.8±0.2	12	93/100	< 0.0001	0.845	< 0.0001	< 0.0001	-	< 0.0001		
isp-1(qm150) - PD	4.6±0.1	4	88/90	< 0.0001	< 0.0001	0.058	< 0.0001	< 0.0001	-		
**panel c**											
				SD+Vehicle	SD+NAC low	SD+NAC high	SD+VitC low	SD+VitC high			
SD+Vehicle	11.4±0.2	11	68/100	-	< 0.0001	< 0.0001	< 0.0001	< 0.0001			
SD+NAC low	13.6±0.2	13	77/100	< 0.0001	-	< 0.001	0.134	0.299			
SD+NAC high	12.5±0.2	13	76/100	< 0.0001	< 0.001	-	< 0.0001	< 0.0001			
SD+VitC low	14.0±0.2	14	86/100	< 0.0001	0.134	< 0.0001	-	0.577			
SD+VitC high	13.8±0.2	13	94/100	< 0.0001	0.299	< 0.0001	0.577	-			
**panel d**											
				BL+Vehicle	BL+VitC low	BL+VitC high	BL+NAC low	BL+NAC high	NL+Vehicle		
BL+Vehicle	11.8±0.3	14	95/100	-	< 0.0001	< 0.0001	< 0.0001	< 0.0001	< 0.0001		
BL+VitC low	16.9±0.3	17	98/100	< 0.0001	-	0.132	< 0.0001	< 0.0001	0.957		
BL+VitC high	16.2±0.4	17	85/89	< 0.0001	0.132	-	< 0.0001	< 0.0001	0.440		
BL+NAC low	14.4±0.4	14	77/89	< 0.0001	< 0.0001	< 0.0001	-	< 0.0001	< 0.01		
BL+NAC high	19.9±0.4	21	97/100	< 0.0001	< 0.0001	< 0.0001	< 0.0001	-	< 0.0001		
NL+Vehicle	15.3±0.5	17	98/100	< 0.0001	0.957	0.440	< 0.01	< 0.0001	-		

Supplementary Table 2: List of strains used in this study

**Strain**	**genotype**
N2	*C. elegans* wild type isolated from Bristol, UK
SJ4100	zcIs13[hsp-6p::GFP])
CL2166	dvIs19[(pAF15)gst-4p::GFP::NLS] III
SJ4103	zcIs14[myo-3::GFP(mit)]
CF1553	muIs84[(pAD76) sod-3p::GFP + rol-6]
SJ4005	zcIs4[hsp-4p::GFP]
JU361	*C. elegans* wild isolate from Franconville, France.
CF1041	*daf-2(e1370)*
CF1085	*daf-2(e1370)xdaf-16(mu86)*
MQ887	*isp-1(qm150) IV*
PR671	*tax-2(p671) I*
PR691	*tax-2(p691) I*
PR694	*tax-2(p694) I*
RB765	*lite-1(ok530) X*
KG1180	*lite-1(ce314) X*
TQ1101	*lite-1(xu7) X*

Supplementary Table 3: List of primers used for QPCR experiments

Transcript	Forward Primer	Reverse Primer
ctl-1	gaatgtgaagaattatttcgctga	aactcgattcctgggacgat
ctl-2	caaggaactacttcgctgagg	aatgagtgtcggtgtacgagaa
ctl-3	gaatgtgaagaattatttcgctga	aactcgattcctgggacgat
gpx-5	gcactttggcagctcacat	ttgactccagcgagtcgttt
gst-4	gatacttggcaagaaaatttggac	ttgatctacaattgaatcagcgtaa
gst-5	gccagcattgaaagaaacct	tttttccgttgagcttgaact
gst-9	caagaatttattgatgagcgtatttct	acttgtccaaatggggtcata
gst-10	actggagcaattatgcgtca	ctccctcgaagaacatgtcg
gst-19	tgattgcccgtttaaagatga	ctgacccattggggacttt
gst-24	ggagcgttgaagccaaaa	gaagccatcgactgagagga
prdx-2	cattccagttctcgctgaca	gttttgtgatgggtcgatga
sod-1	ctcatggtggaccaaaatcc	cgtgtcggtgagcttgatt
sod-2	ttgttcaaccgatcacagga	gtaaatctggcagcgagtgc
sod-3	cactgcttcaaagcttgttca	atgggagatctgggagagtg
sod-4	cagaccaatcaaaaacaactgg	tgagacgcagacagtgagaca
sod-5	cacacttcaatccatgcaaaa	acgtttccaaggtcaccaac
cup-4	acactgagttttggctggga	gtgctgagcttgagacgttt
fmo-2	atagtggaatcgacgtggca	gagtgagaagaaacgcgtgt
nlp-7	tccgctattgagtcagagcc	tgtcatcaatctcgggtcgt
abu-6	tcgtgccaacaagttcaaca	cgcattgagtagattgaacctga
hsp-6	aaccggaaaggaacaacagat	tctcgatttggtccttggaa
hsp-60	agggattcgagagcattcgtcaag	tgtggcgacttgagcgatctcttc
hsp-4	cagatgaaaactcaaatcgcc	ggttgcttccgagccactcaa
xbp-1s	cgtgcctttgaatcagcagtg	cgaggtgtccatcttcttgtt
hsp-70a	cggtatttatcaaaatggaaaggtt	tacgagcggcttgatctttt
hsp-16.2	tgcagaatctctccatctgagt	tggtttaaactgtgagacgttga
hsp-12.3	ggccattccagaaaggaga	caatgttcttcacatcaatctcg
hsp-16.1	ggctcagatggaacgtcaa	tggcaaacttttgatcattgtta
hsp-16.11	ggctcagatggaacgtcaa	tggcaaacttttgatcattgtta

hsp-16.41	tcttggacgaactcactgga	agagacatcgagttgaaccga
hsp-16.49	ctcatgctccgttctccatt	gagttgtgatcagcatttctcca
cdc-42	aggaacgtcttccttgtctcc	ggacatagaaagaaaaacacagtcac
pmp-3	cggtgttaaaactcactggaga	tcgtgaagttccataacacga
y45f10d.4	aagcgtcggaacaggaatc	tttttccgttatcgtcgactc
